# Dosing, Safety, and Tolerability of Extended‐Release Tacrolimus in Pediatric and Young Adult Solid Organ Transplant Recipients

**DOI:** 10.1111/petr.70349

**Published:** 2026-05-23

**Authors:** Terry Dang, Jennifer Hewlett, John McAteer, Kevin J. Downes

**Affiliations:** ^1^ Clinical Pharmacology Postdoctoral Training Program Children's Hospital of Philadelphia Philadelphia Pennsylvania USA; ^2^ Department of Pediatrics Children's Hospital of Philadelphia Philadelphia Pennsylvania USA; ^3^ Division of Nephrology Children's Hospital of Philadelphia Philadelphia Pennsylvania USA; ^4^ Division of Infectious Diseases Children's Hospital of Philadelphia Philadelphia Pennsylvania USA; ^5^ Department of Pediatrics Perelman School of Medicine of the University of Pennsylvania Philadelphia Pennsylvania USA

## Abstract

**Background:**

Complicated immunosuppression regimens can contribute to non‐adherence in pediatric solid organ transplant (SOT) recipients. Once‐daily tacrolimus formulations, such as LCP‐tacrolimus (LCPT), may help simplify dosing, but pediatric data on dosing, safety, and early tolerability are limited.

**Methods:**

We conducted a single center retrospective cohort study of SOT recipients prescribed LCPT from December 2016 to July 2024 at our institution. LCPT:IR‐Tac daily dosing ratios were calculated. The frequency of laboratory‐associated adverse events (AE) and of new patient‐reported adverse events in the first 90 days were collected and compared across pediatric‐ and adult‐aged SOT recipients.

**Results:**

Ninety‐one SOT recipients were prescribed LCPT at a median age of 16.8 years (range 8.4–24.1) and 75 months after transplant (range 0.25–217). Among 53 patients who were therapeutic on both IR‐Tac and LCPT, the median daily dosing ratio of LCPT:IR‐Tac was 0.75 (range 0.33–1.5) for pediatric patients and 0.75 (IQR: 0.46–1.33) for adults. Eleven (5 patient‐reported and 6 laboratory) AEs were identified, none of which resulted in cessation of LCPT therapy. There were no significant differences in the frequency of AEs between children and adults.

**Conclusions:**

LCPT was well tolerated and typically required about a 25% dose reduction compared with IR‐tacrolimus, though conversion ratios varied widely. These findings offer practical guidance for LCPT dosing in pediatric transplant care and highlight the need for individualized titration and monitoring.

## Graphical abstract

Extended‐release tacrolimus (LCPT) was safe and well‐tolerated among 91 children and young adult solid organ transplant patients at our hospital. However, the dosing ratio of LCPT:immediate‐release tacrolimus (mg/day) varied widely across patients, highlighting the importance of close therapeutic drug monitoring when transitioning to LCPT.

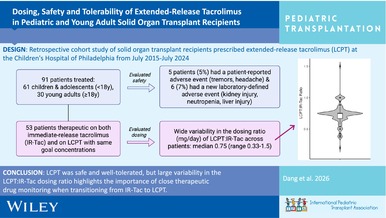

**Abbreviations**
95% CI: 95% confidence interval
AKI: acute kidney injury
ALP: alkaline phosphatase
ALT: alanine aminotransferase
ANC: absolute neutrophil count
AST: aspartate aminotransferase
AUC: area under the curve
CYP: Cytochrome P450
DILI: drug‐induced liver injury
IQR: inter‐quartile range
IR‐Tac: immediate release tacrolimus
LCPT: Life Cycle Pharma Tacrolimus
PK: pharmacokinetics
SOT: solid organ transplant
ULN: upper limit of normal

## 1 Introduction

Tacrolimus, a calcineurin inhibitor (CNI), is a mainstay of solid organ transplant (SOT) immune suppression. Daily administration of this agent is used to prevent rejection in most pediatric SOT (pSOT) recipients. Inconsistent adherence to immunosuppressant regimens can result in significant negative outcomes following transplantation [1]. Children with chronic conditions requiring strict medication adherence, including SOT, have reported rates of adherence as low as 5%–15% attributed to a lack of understanding about the disease or treatment, culture, socioeconomic status, family structure, administration schedule, pill burden, and taste. Thus, identification of approaches that simplify post‐transplant care are important. Individualized strategies that target interventions to patients and families are most effective in promoting long term adherence [2].

Tacrolimus is available in four commercially available formulations: an immediate‐release, twice‐daily formulation given as tablet(s) or granules (IR‐Tac; Prograf; Astellas Pharma US Inc); a prolonged‐release, once‐daily formulation (PR‐Tac; Astagraf XL; Astellas Pharma US Inc); and an extended‐release once‐daily formulation (LCPT; Envarsus XR, Veloxis Pharmaceuticals Inc., Edison NJ). Younger pSOT recipients often receive a compounded suspension of IR‐Tac due to an inability to tolerate solid dosage forms.

IR‐Tac introduces several challenges for pSOT including large intra‐ and inter‐patient pharmacokinetic (PK) variability related to absorption, disposition and clearance, as well as age‐dependent CYP3A enzyme maturation, which significantly affects tacrolimus metabolism and concentrations [3, 4]. Frequent therapeutic monitoring of serum tacrolimus concentrations is necessary due to tacrolimus's narrow therapeutic index to mitigate the risk of significant complications including adverse events which may impact allograft function [5, 6]. Twice‐daily dosing of IR‐Tac also contributes to increased pill burden and non‐adherence, particularly in adolescents and young adults.

LCPT was developed to increase the solubility and bioavailability of tacrolimus, enabling once‐daily administration [7]. In adult SOT recipients, LCPT demonstrates greater exposure per milligram of dose, reduced peak‐to‐trough concentration ratios, and a more sustained absorption profile compared with immediate‐release tacrolimus (IR‐Tac) and prolonged‐release tacrolimus (ER‐Tac) formulations [8, 9]. Additionally, decreased fluctuations in tacrolimus concentrations with LCPT may contribute to improved tolerability and adherence as compared to traditional tacrolimus formulations [9, 10]. Current experience with LCPT in children and adolescents is limited. A single‐center study of 29 adolescent and young adult SOT recipients found that conversion to LCPT was associated with numeric but not statistically significant improvement in medication adherence with no differences in episodes of rejection or adverse effects [11]. Another retrospective study of 41 pediatric and young adults kidney and liver transplant recipients reported similar rates of acute kidney injury (AKI) and rejection before and after conversion from IR‐Tac to LCPT [12]. Meanwhile, a retrospective study of 29 adolescent and young adult SOT recipients reported reduced pill burden and improved variability in tacrolimus trough concentrations [13].

Given the limited data available on LCPT use in pSOT recipients, we sought to describe the use of LCPT at our institution. The primary objectives were to derive conversion ratios of LCPT:IR‐Tac in adult and pSOT recipients and to evaluate short‐term safety and tolerability within our SOT population.

## 2 Patients and Methods

### 2.1 Study Design

We conducted a retrospective study of all SOT recipients administered LCPT at the Children's Hospital of Philadelphia (CHOP) from July 2015 through July 2024. Patients were included if they had a history of SOT and received ≥ 1 prescription for LCPT by a CHOP provider. Patients were excluded if they were ordered but did not receive LCPT or if they had no follow‐up at CHOP after LCPT was initiated. During the time period of the study, decisions to start LCPT were made by transplant providers with input from a transplant clinical pharmacist. Conversion from IR‐Tac to LCPT generally followed the product labeling recommendations in which LCPT was prescribed at a daily dose of 80% of the total daily dose of IR‐Tac, although specific dosing was at their discretion.

Demographic, transplant history, prior and concurrent medications, indication for LCPT conversion/initiation, laboratory results, and new adverse events were collected from electronic medical records via automated and manual data collection. Manual chart review of clinical encounters within the 90 days before and after initiation of LCPT was performed. All data were stored using Research Electronic Data Capture (REDCap) [14]. The study was determined to meet the exemption criteria per 45 CFR 46.104(d) 4(iii) by the CHOP Institutional Review Board (IRB 23‐021817).

### 2.2 Definitions

Children and adolescents were classified as being pediatric (< 18 years of age) while those ≥ 18 years of age at the time of starting LCPT were categorized as adults. The indications for starting LCPT were classified as adherence issues, toxicity/adverse effects of IR‐Tac, or other/unspecified indication; this latter category was used for all individuals in whom adherence or toxicity issues were not specifically documented as an indication for LCPT and included instances where the rationale was to simplify the dosing regimen, as well as cases without clear documentation regarding the reason for switching.

The therapeutic target trough range for each individual was defined based on provider documentation. A tacrolimus concentration was deemed to be therapeutic if it was within the therapeutic range and there was no documentation of concerns (e.g., sample mistimed) by a clinical provider. Patients were defined as being therapeutic on IR‐Tac and/or on LCPT if they had ≥ 1 tacrolimus level that was therapeutic in the 90 days prior to starting LCPT or following the start of LCPT, respectively.

The daily dosing ratio of LCPT:IR‐Tac was derived for each individual who transitioned directly from IR‐Tac to LCPT; patients transitioning from other extended‐release tacrolimus formulations to LCPT or starting LCPT *de novo* were not included in this analysis. The initial LCPT:IR‐Tac ratio was calculated using the daily dose (mg) of IR‐Tac at transition to LCPT and the daily dose of LCPT first prescribed regardless of serum tacrolimus concentrations. We then identified the subset of individuals who (a) had the same therapeutic tacrolimus trough target range on IR‐Tac and LCPT and (b) were therapeutic on both IR‐Tac at transition to LCPT and on LCPT within the first 90 days of initiation. We calculated the LCPT:IR‐Tac ratio (therapeutic dosing ratio) for this subset using the therapeutic daily dose (mg) of IR‐Tac closest to transition to LCPT and the daily dose of LCPT that was first therapeutic.

Patient‐reported adverse events included tremor, headache, sleep disturbance, and visual disturbance. These were classified as pre‐existing if documented in a clinical encounter progress note in the 90 days prior to starting LCPT or new onset if first reported in the 90 days after LCPT initiation. The relationship between a new‐onset patient‐reported adverse event and LCPT was characterized as possibly related if the symptom(s) improved following a dosing modification or stoppage of LCPT.

Laboratory‐associated adverse events in the first 90 days after starting LCPT were captured among individuals who had sufficient laboratory testing performed to characterize these events. Acute kidney injury (AKI) was defined as a ≥ 50% increase in serum creatinine from baseline within 7 days on ≥ 2 consecutive measurements, according to KDIGO criteria [15]; baseline creatinine was defined as the lowest creatinine measurement in the 90 days preceding LCPT start. Drug‐induced liver injury (DILI) was defined as having at least one of the following: (1) serum aspartate aminotransferase (AST) or alanine aminotransferase (ALT) > 5 times upper limit of normal (ULN) or alkaline phosphatase (ALP) > 2 times ULN (or the baseline value if baseline is elevated) on two consecutive occasions, (2) total serum bilirubin > 2.5 mg/dL with elevated AST, ALT or ALP, or (3) international normalized ratio > 1.5 with elevated AST, ALT or ALP [16]; baseline was defined as the measurement closest to the start of LCPT initiation. Neutropenia was defined as an absolute neutrophil count (ANC) < 1000 cells/hpf on two consecutive measurements; only individuals with an ANC > 1500 cells/hpf at LCPT initiation were eligible for this analysis.

### 2.3 Statistical Analyses

Continuous variables were described using medians with ranges; inter‐quartile range (IQR) was also provided for LCPT:IR‐Tac ratios. LCPT:IR‐Tac ratios were summarized by age group (pediatric [< 18 years], adult) and race (White, Black, Other); CYP genotype data were not performed clinically and thus not available. Categorical variables were summarized using frequencies or proportions. For group comparisons (e.g., pediatric vs. adult SOT recipients; therapeutic vs. not therapeutic on LCPT), Wilcoxon rank sum tests and chi‐squared (or Fisher's exact) tests were utilized to compare continuous data and categorical data, respectively. Statistical analyses were performed using R v4.5.0 (The R Foundation, Vienna, Austria).

## 3 Results

**FIGURE 1:**
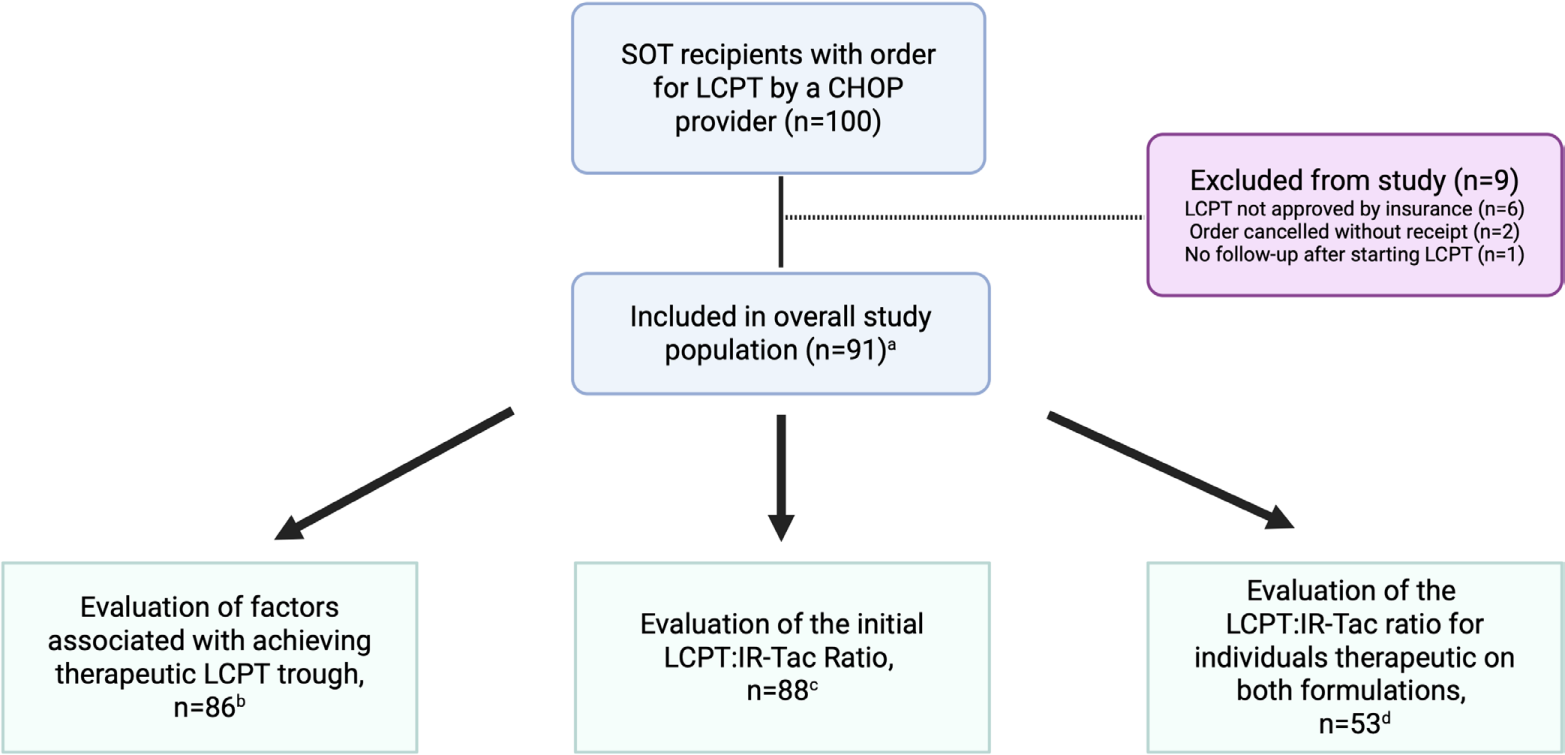
Flow diagram of study cohort and analyses. ^a^Primary study population used for descriptive analyses and assessment of patient‐reported and laboratory‐associated adverse events. ^b^Subgroup analysis included patients with a consistent trough goal range over the first 90 days of LCPT. ^c^Subgroup analysis included individuals transitioned directly from IR‐Tac to LCPT; excluded 3 patients who transitioned from another prolonged‐release formulation (*n* = 2) or from everolimus (*n* = 1) to LCPT. ^d^Subgroup analysis included individuals therapeutic on both IR‐Tac and LCPT; excluded 35 patients who were not therapeutic on IR‐Tac at transition (*n* = 13), who were not therapeutic on LCPT within 90 days (*n* = 20), whose target trough differed between IR‐Tac and LCPT (*n* = 5), or who did not have clear target trough goal in the EMR (*n* = 5). Exclusions were not mutually exclusive. CHOP, Children's Hospital of Philadelphia; IR‐Tac, immediate‐release tacrolimus; LCPT, extended‐release tacrolimus. Figure made using Biorender.com.

In total, 100 patients were prescribed LCPT by a CHOP provider (Figure 1) during our study period. Nine patients were excluded from our analysis: 6 patients were denied LCPT by insurance, 2 had their LCPT order canceled prior to receipt, and 1 transferred care to another institution without any follow‐up at CHOP. Thus, 91 patients comprised our final study cohort, including 61 children and 30 adults (Table 1). No patients were prescribed de‐novo LCPT immediately following transplantation. The median time to LCPT initiation following transplant was 75 months (range: 0.25–217), with most patients (*n* = 80, 88%) prescribed LCPT as an outpatient. The primary indication for starting LCPT was to simplify the IS regimen in 53 (58%) patients followed by improvement in medication adherence (*n* = 25, 27%) and IR‐Tac toxicity (*n* = 13, 14%).

**TABLE 1:** Characteristics of study population.

	*n* = 91
Age at the time transition to LCPT in years, median (range)	16.8 (8.4–24.1)
Adults ≥ 18 years, *n* (%)	30 (33)
Adolescents 12–17 years, *n* (%)	51 (56)
Children < 12 years, *n* (%)	10 (11)
Female sex, *n* (%)	41 (45)
Race, *n* (%)	
Black/African	21 (23)
Caucasian	43 (47)
Asian	3 (3)
Pacific Islander	1 (1)
Other/Unknown/Not reported	23 (25)
Hispanic ethnicity, *n* (%)	19 (21)
Organ transplanted, *n* (%)^a^	
Liver	42 (46)
Kidney	32 (35)
Lung	16 (18)
Heart	4 (4)
Time (months) from transplant to starting LCPT, median (range)	75 (0.25–217)
Indication for starting LCPT, *n* (%)	
Adherence issues	25 (27)
Toxicity/adverse effect from IR‐Tac	13 (14)
Other/unspecified indication	53 (58)
Other immune suppression at transition to LCPT,*n* (%)	
Mycophenolate mofetil (MMF)	40 (44)
Steroids	46 (51)
Sirolimus	3 (3)
Azathioprine	11 (12)
None (tacrolimus monotherapy)	37 (41)
Therapeutic target for LCPT, in ng/mL^b^, ^c^	
≤ 3	22 (26)
3–5	4 (5)
4–5	3 (3)
4–6	16 (19)
5–8	4 (5)
6–8	17 (20)
8–10	6 (7)
8–12	4 (5)
Other	10 (12)

^a^ Includes two patients who had received a kidney and a liver transplant and 1 patient who had received a heart and a kidney transplant.

^b^ Reported for 86 patients with consistent LCPT targets.

^c^ Categories displayed if reported for ≥ 3 patients.

Of the 91 patients prescribed LCPT, 86 had a consistent therapeutic goal range over the entirety of their first 90 days after starting the drug, regardless of whether they were therapeutic on IR‐Tac. Twenty patients (23%) failed to achieve a therapeutic level in this 90‐day period. Patients who failed to achieve a therapeutic level in the first 90 days were significantly farther out from transplant (median 111.0 vs. 62.7 months, Wilcoxon rank *p* = 0.03). There were no differences in age among those who did and did not have ≥ 1 therapeutic tacrolimus level within the first 90 days (Wilcoxon rank *p* = 0.85). Kidney transplant recipients most often achieved a therapeutic level in this time period (*n* = 26/29; 90%), followed by liver (*n* = 28/38; 74%) and lung (*n* = 9/15; 60%; *p* = 0.07); both heart transplant recipients were therapeutic within 90 days. Of the 66 patients with a therapeutic level in the first 90 days of LCPT, most required 0 (*n* = 40) or 1 (*n* = 19) dose adjustment to achieve their therapeutic target, while 7 required 2 or more dose adjustments. The proportion of patients requiring 2 or more dose adjustments was similar among the 3 indication groups (*p* = 0.63); age was also similar among patients who did and did not require 2 or more dose adjustments to achieve a therapeutic target within 90 days (*p* = 0.18).

Among the cohort, 88 patients transitioned directly from IR‐Tac to LCPT; 3 were on some other form of IS prior to starting LCPT. The median LCPT:IR‐Tac daily dosing ratio at transition was 0.75, although the ratio ranged from 0.2 to 1.5 across patients.

**FIGURE 2:**
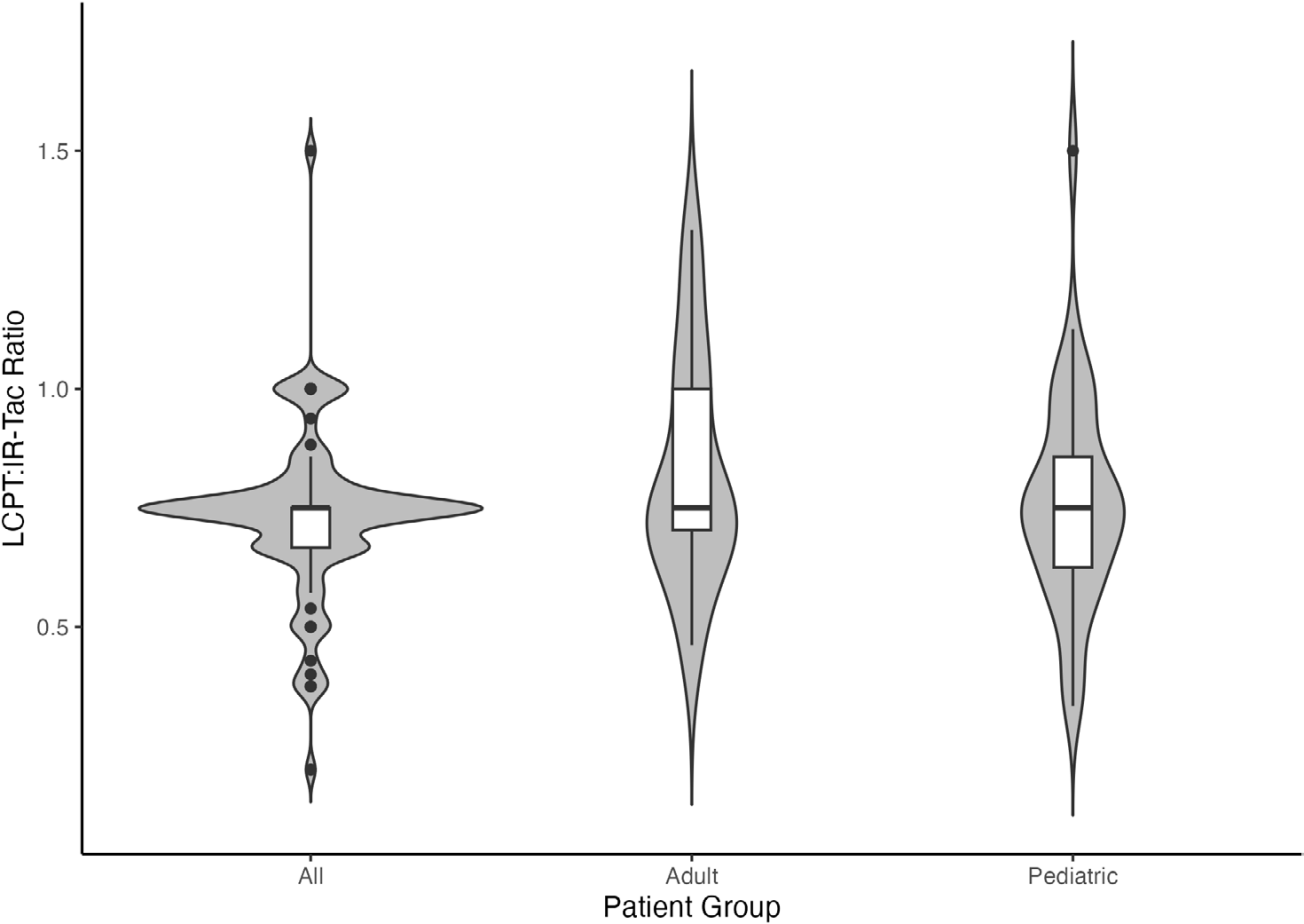
Violin plots of LCPT:IR‐Tac ratio among patients therapeutic on both IR‐Tac and LCPT. Dots represent ratios for adult (*n* = 18) and pediatric (*n* = 35) patients. Overlaid boxplots show median (thick black line), interquartile range (IQR; white boxes), and range of the data (lines span from 1st quartile—1.5 × IQR).

Of patients transitioned from IR‐Tac to LCPT, 53 (60%) were therapeutic on IR‐Tac at the time of transition and established a therapeutic level within 90 days of starting LCPT (Figure 2), including 18 adults and 35 children. The median LCPT:IR‐Tac ratio among patients who were therapeutic on both IR‐Tac and LCPT was 0.75 (range 0.33–1.5; IQR 0.67–0.92). This ratio was the same for pediatric (median 0.75, range 0.33–1.5; IQR 0.63–0.86) and adult patients (median 0.75, range 0.46–1.33; IQR 0.70–1.0). The median LCPT:IR‐Tac ratio for each of the three race groups (White, Black, Other) was also 0.75 (White: *n* = 26, range: 0.33–1.5, IQR: 0.64–0.81; Black: *n* = 12, range: 0.4–1.0, IQR: 0.66–0.86; Other: *n* = 12, range: 0.38–1.33, IQR: 0.66–1.0).

There were few patient‐reported or laboratory‐associated adverse events over the first 90 days after starting LCPT (Table 2). In total, 5 patients had documented ≥ 1 patient‐reported adverse event, including 2 (3.3%) pediatric patients and 3 (10%) adults (Fisher's exact *p* = 0.33). Meanwhile, 6 patients experienced a laboratory‐defined adverse event in the first 90 days after starting LCPT: 3 episodes of AKI, 2 of neutropenia, and 1 of liver injury (hyperbilirubinemia with elevated liver enzymes). All six of the laboratory‐associated adverse events occurred in children (Fisher's exact *p* = 0.17, compared to adults). None of the patient‐reported or laboratory‐associated adverse events were ascribed to LCPT by the clinical team nor led to discontinuation or dose modifications of the drug.

**TABLE 2:** Patient‐reported and laboratory‐defined adverse events in the first 90 days after transitioning to LCPT.

	*n*/*N* (%)
Patient‐reported adverse events^a^	
Tremors	3/69 (4.3)
Headaches	2/73 (2.7)
Sleep disturbance	0/81 (0)
Visual disturbance	0/90 (0)
Laboratory‐defined adverse events	
Acute kidney injury^b^	3/83 (3.6)
Neutropenia^c^	2/82 (2.4)
Drug‐induced liver injury^c^	1/83 (1.2)

^a^ Numerator for determination of percentages is patients with new‐onset symptoms and the denominator is based on the number of patients who did not have pre‐existing symptoms.

^b^ Denominator for determination of percentages based on individuals with a baseline measurement (within 90 days prior to starting LCPT) and ≥ 1 measurement within 90 days of LCPT start.

^c^ Denominator for determination of percentages based on individuals with ≥ 1 measurement within 90 days of LCPT start.

## 4 Discussion

Tacrolimus is an integral component of post‐transplant immunosuppression in SOT. Though currently only approved for use in adult transplant recipients, LCPT has been increasingly prescribed off label at pediatric transplant centers like ours. As with other, smaller single‐center reports [11, 12, 13, 17] we found that LCPT was safe and well‐tolerated among the 61 pediatric and 30 adult SOT recipients at our institution. Though there were numerically more laboratory‐associated adverse events in children prescribed LCPT, we found no statistically significant differences in the frequency of patient‐reported or laboratory‐associated adverse events in the first 90 days following conversion to LCPT between pediatric and adult SOT recipients.

A potential benefit of LCPT compared to IR‐Tac is that it is administered once daily. This can simplify the medication regimen for adolescents and young adults learning to manage their own post‐transplant care. One quarter of patients transitioned to LCPT at our center did so because of adherence issues with IR‐Tac. Though we did not have a reliable way to assess adherence after transitioning to LCPT since our study was retrospective, we did not identify any patients who switched back to IR‐Tac or to another agent due to documented non‐adherence. Interestingly, however, nearly a quarter (23%) of patients failed to achieve a therapeutic tacrolimus level in the first 90 days, suggesting that adherence may still be challenging for some patients and families even when the tacrolimus regimen is simplified. Alternatively, because patients who failed to attain a therapeutic level on LCPT were nearly 10 years post‐transplant, on average, TDM may have been performed less often, or teams may have been less strict about ensuring that therapeutic levels were achieved. Since missed doses could have more significant immunologic consequences when dosing is only once daily, providers should incorporate counseling into all tacrolimus formulation transition discussions.

Once‐daily dosing can reduce pill burden, which is particularly relevant in pediatric and adolescent transplant populations in whom non‐adherence is a recognized contributor to graft dysfunction and rejection. Improved adherence could potentially translate into fewer rejection episodes, hospitalizations, and graft‐related complications, all of which carry substantial healthcare costs. Further, for families that struggle to reliably obtain tacrolimus concentrations, LCPT may reduce the frequency of therapeutic drug monitoring, potentially lowering laboratory and clinical management costs over time. Among patients who achieved their therapeutic target on LPCT within 90 days at our center, most (89%) did so with 1 or fewer dose adjustments. However, the overall cost‐effectiveness of switching to LCPT remains dependent on institutional pricing, insurance coverage, and patient‐specific factors. In many healthcare settings, the higher direct medication cost remains a limiting factor for widespread adoption. Therefore, economic evaluations comparing medication costs with potential downstream savings related to improved adherence, reduced toxicity, and fewer graft‐related complications would be valuable.

Though safe and well tolerated in the short‐term, we did not evaluate the incidence of rejection among our study population given the retrospective nature of the study. Other cohorts of adolescents and young adults have reported a similar incidence rate of rejection pre‐ and post‐conversion to LCPT [11, 12, 13]. Our population was heterogeneous in terms of timing of LCPT conversion post‐transplant and the degree of concurrent immune suppression at LCPT start. Thus, we did not feel that we could reliably interpret how LCPT impacts organ rejection. However, prospective studies and clinical trials are needed to better understand LCPT's role in pediatric SOT care. No patients in our study population began LCPT as part of their initial immune suppression regimen post‐transplant, but increased experience and availability of data on LCPT's PK and long‐term safety may promote earlier use in the pediatric transplant.

IR‐Tac is rapidly absorbed in the proximal small intestine, where extensive first‐pass metabolism contributes to higher peak concentrations, greater peak‐to‐trough variability, and increased intra‐patient pharmacokinetic variability. However, once‐daily LCPT utilizes MeltDose technology to enhance oral bioavailability, resulting in delayed time to peak concentration and more distal intestinal absorption. As a result, LCPT achieves comparable systemic exposure at a lower total daily dose, with a flatter concentration–time profile and reduced peak‐to‐trough fluctuations. Given these formulation‐specific differences in sites of intestinal absorption, gastrointestinal illness may differentially affect tacrolimus absorption and exposure between IR‐Tac and LCPT, representing an important area for future investigation.

A pharmacological advantage of LCPT compared to IR‐Tac is that its longer half‐life allows for decreased variability in peak and trough concentrations over the course of the day. And, due to its increased bioavailability compared to other tacrolimus formulations, a lower daily dose of LCPT is typically needed compared to IR‐Tac. According to the package insert, for adult kidney transplant recipients converting from IR‐Tac to LCPT, it is recommended to administer a dose of LPCT that is 80% of the IR‐Tac daily dose [7]. We found that the median LCPT:IR‐Tac dosing ratio was 0.75 for both children and adults, which is similar to other reports [12]. Yet, there was a very wide range in the dosing ratios across individuals (0.33–1.5). Interpatient pharmacokinetic variability, differences in hepatic function, potential drug–drug interactions, age‐related metabolic differences, and pharmacogenetic factors such as CYP3A5 expression, all may have contributed to the wide range of dosing ratios seen in our study. Therefore, monitoring of trough concentrations is still imperative. Importantly, measurement of trough concentrations is a surrogate for overall tacrolimus exposure (i.e., 24‐h area‐under‐the‐curve) and with limited pediatric PK data available, it's uncertain if the same trough targets should be used for LCPT as for IR‐Tac in pediatric SOT recipients, although that is what is routinely done. Additional research in this area is urgently needed.

Furthermore, tacrolimus is primarily metabolized by CYP3A5 enzymes in the liver and gut [18] The Clinical Pharmacogenetics Implementation Consortium (CPIC) recommends the use of 1.5–2 times increased starting doses for intermediate and extensive metabolizers [19] However, the implications of pharmacogenetic variations on tacrolimus metabolism and concentrations for different formulations are not fully understood. Pharmacogenetic testing was not routinely performed at our center and thus was not available to us. Although our population was largely Caucasian, variability in CYP3A5 expression may have contributed to the wide range of LCPT:IR‐Tac ratios seen.

There are important limitations to our study, mostly related to its retrospective design and risk of ascertainment bias. We focused on safety and tolerability in the 90 days after initiation of LCPT knowing that the majority of SOT care post‐transplant is in the outpatient setting. Since our study was retrospective, we believed that laboratory testing and follow‐up would be most rigorous in the first few weeks after starting LCPT. Despite this, it's possible that adverse events were not fully captured due to inadequate documentation in the medical record or because labs were not performed at sufficient intervals to capture transient abnormalities. Further, we cannot attribute any identified laboratory events to receipt of LCPT, specifically. While temporally associated with initiation of LCPT, we do not know how often the few adverse that occurred were directly related to the medication. Fortunately, we reviewed documentation associated with every LCPT dosing change and did not identify any adverse events that led to changes in LCPT. Furthermore, by focusing on short‐term adverse events our study was not designed to evaluate longer term outcomes that are pertinent to SOT care (e.g., rejection, graft function). Larger, controlled studies would be necessary to determine the comparative effectiveness of LCPT to other tacrolimus formulations and to other immunosuppressive agents. Lastly, we compared attainment of target trough concentrations only, which are what is measured clinically at our center, rather than full AUCs. Additional studies are needed to better understand how overall tacrolimus exposure profiles differ among children and young adults treated with LCPT compared to IR‐Tac.

In summary, our study is the largest single center evaluation of LCPT use in pediatric patients to date. Our findings confirm that a daily dosing ratio of 0.75 for LCPT:IR‐Tac is reasonable for most SOT recipients, regardless of age. LCPT was well‐tolerated among our cohort, but close monitoring of tacrolimus concentrations is warranted.

## Funding

This work was supported by Veloxis Pharmaceuticals. National Institute of General Medical Sciences, T32GM008562.

## Data Availability

The data that support the findings of this study are available on request from the corresponding author. The data are not publicly available due to privacy or ethical restrictions.
